# Urinary Bisphenol A Concentrations and Implantation Failure among Women Undergoing *in Vitro* Fertilization

**DOI:** 10.1289/ehp.1104307

**Published:** 2012-04-06

**Authors:** Shelley Ehrlich, Paige L. Williams, Stacey A. Missmer, Jodi A. Flaws, Katharine F. Berry, Antonia M. Calafat, Xiaoyun Ye, John C. Petrozza, Diane Wright, Russ Hauser

**Affiliations:** 1 Department of Environmental Health, and; 2 Department of Biostatistics, Harvard School of Public Health, Boston, Massachusetts, USA; 3 Department of Obstetrics, Gynecology, and Reproductive Biology, Brigham and Women’s Hospital and Harvard Medical School, Boston, Massachusetts, USA; 4 Department of Epidemiology, Harvard School of Public Health, Boston, Massachusetts, USA; 5 Comparative Biosciences, University of Illinois at Urbana–Champaign, Urbana, Illinois, USA; 6 National Center for Environmental Health, Centers for Disease Control and Prevention, Atlanta, Georgia, USA; 7 Vincent Memorial Obstetrics and Gynecology Service, Massachusetts General Hospital, Boston, Massachusetts, USA

**Keywords:** bisphenol A, embryo implantation, fertility, human, reproduction, women

## Abstract

Background: Bisphenol A (BPA) is a synthetic chemical widely used in the production of polycarbonate plastic and epoxy resins found in numerous consumer products. In experimental animals, BPA increases embryo implantation failure and reduces litter size.

Objective: We evaluated the association of urinary BPA concentrations with implantation failure among women undergoing *in vitro* fertilization (IVF).

Methods: We used online solid phase extraction–high performance liquid chromatography–isotope dilution tandem mass spectrometry to measure urinary BPA concentrations in 137 women in a prospective cohort study among women undergoing IVF at the Massachusetts General Hospital Fertility Center in Boston, Massachusetts. We used logistic regression to evaluate the association of cycle-specific urinary BPA concentrations with implantation failure, accounting for correlation among multiple IVF cycles in the same woman using generalized estimating equations. Implantation failure was defined as a negative serum β-human chorionic gonadotropin test (β-hCG < 6 IU/L) 17 days after egg retrieval.

Results: Among 137 women undergoing 180 IVF cycles, urinary BPA concentrations had a geometric mean (SD) of 1.53 (2.22) µg/L. Overall, 42% (*n* = 75) of the IVF cycles resulted in implantation failure. In adjusted models, there was an increased odds of implantation failure with higher quartiles of urinary BPA concentrations {odds ratio (OR) 1.02 [95% confidence interval (CI): 0.35, 2.95}, 1.60 (95% CI: 0.70, 3.78), and 2.11 (95% CI: 0.84, 5.31) for quartiles 2, 3, and 4, respectively, compared with the lowest quartile (*p*-trend = 0.06).

Conclusion: There was a positive linear dose–response association between BPA urinary concentrations and implantation failure.

Bisphenol A (BPA) is a ubiquitous chemical widely used in the manufacture of polycarbonate plastics found in some water bottles (Brede et al. 2003), the lining of food and beverage cans and water pipes (Bae et al. 2002), and some dental sealants and composites (Joskow et al. 2006; Sasaki et al. 2005). BPA can also be used in the manufacture of thermal receipt paper (Biedermann et al. 2010; vom Saal and Myers 2008). Widespread use of BPA-containing consumer products has led to ubiquitous exposure to BPA in the general population (Vandenberg et al. 2007). In the 2003–2004 National Health and Nutrition Examination Survey (NHANES), BPA was detected in > 90% of urine samples obtained from a representative sample of U.S. residents (Calafat et al. 2008). Detectable concentrations of BPA have also been measured in human follicular fluid (1–2 µg/L) and amniotic fluid (1–9 µg/L) (Ikezuki et al. 2002), suggesting that exposure may occur as early as the periconception period.

During the past several decades, infertility rates have increased, and approximately 10–15% of couples in the United States and other developed countries are infertile (Fritz and Speroff 2011), in part because of delayed childbearing. An important determinant of reduced fertility is failed implantation, which is thought to account for 50–75% of preclinical pregnancy losses in humans (Macklon et al. 2002; Norwitz et al. 2001). Implantation is orchestrated and regulated by a very carefully synchronized interplay of hormonal signals and feedback loops, making it potentially vulnerable to chemicals such as BPA that may disrupt endocrine signaling (Berger et al. 2010; Ma et al. 2003; Varayoud et al. 2011).

Although BPA has been known to have estrogenic properties since 1936 (Dodds and Lawson 1936), the biological activity of BPA is rather complex and still not fully understood. More recently in *in vitro* studies, BPA was found to have measurable activity in multiple assays involving signaling pathways for estrogen, androgen, and thyroid hormones (Food and Agriculture Organization of the United Nations / World Health Organization 2010; Lee et al. 2003; Quesada et al. 2002; Welshons et al. 2006; Wozniak et al. 2005). These hormonal effects may contribute to a disruption of reproductive function. In addition, experimental studies in animals suggest that BPA exposure adversely affects female fertility (Berger et al. 2007, 2008, 2010; Takai et al. 2001; Tsutsui et al. 1998). An increased incidence of failed intrauterine implantation was observed in mice after subcutaneous exposure to environmentally relevant low concentrations of BPA (Takai et al. 2001; Tsutsui et al. 1998). Litter size, number of implantation sites, and the percentage of females delivering pups were decreased after single-dose administration of BPA during the critical time window for implantation. Similar effects were observed in mice after ingestion and subcutaneous administration of higher doses of BPA (Berger et al. 2007, 2008, 2010; Xiao et al. 2011).

Few human studies have examined the association of BPA with female fertility and pregnancy outcomes, primarily because of the practical challenges involved in studying early pregnancy end points among couples conceiving naturally (Fujimoto et al. 2011; Mok-Lin et al. 2010; Sugiura-Ogasawara et al. 2005). Therefore, we investigated the relationship between urinary BPA concentrations and implantation failure among women undergoing *in vitro* fertilization (IVF).

## Methods

*Study participants and data collection.* The present analysis of 137 women is part of a larger prospective cohort study designed to investigate the impact of environmental chemicals on fertility and pregnancy outcomes among couples seeking fertility treatment. All women in the larger study who underwent at least one IVF cycle and had urinary BPA analyzed were included in the present analysis. Study participants were female partners of couples seeking infertility evaluation and treatment at the Massachusetts General Hospital (MGH) Fertility Center, Boston, Massachusetts. Couples were recruited between November 2004 and April 2010. Women 18–45 years of age who used their own oocytes (eggs) for IVF were eligible. The women were followed from study entry through each of their IVF cycles until they had a live birth or discontinued treatment at the MGH Fertility Center. The study was approved by the Institutional Review Boards of the Massachusetts General Hospital, Harvard School of Public Health (HSPH), and the Centers for Disease Control and Prevention (CDC). All participants provided an informed consent after the study procedures were explained by a research nurse and all questions were answered.

At recruitment, a brief, nurse-administered questionnaire was used to collect data on demographics, medical history, and lifestyle. Women also completed a detailed take-home questionnaire with additional questions on lifestyle factors, occupation, and medical history (completed by > 90% of participants). Clinical information was obtained from the electronic medical record, and infertility diagnoses were classified according to the Society for Assisted Reproductive Technology (SART) definitions.

*Treatment protocols and clinical IVF measures*. All women were initially treated with a cycle of oral contraceptive pills (OCP) to suppress ovulation unless it was contraindicated. The day of OCP-induced menses was referred to as cycle day 1 of the treatment cycle, the day after was cycle day 2 and so on. The patient was then monitored at cycle day 3 at the clinic to ensure ovarian suppression before beginning controlled ovarian stimulation with follicle-stimulating hormone (FSH) and gonadotropin-releasing hormone (GnRH) agonists or antagonists. Patients were monitored as needed during gonadotropin stimulation for serum estradiol (E2), follicle size measurements, follicle count, and endometrial thickness through 2 days before the egg-retrieval procedure. Human chorionic gonadotropin (hCG), a hormone similar to luteinizing hormone (LH), was administered (hCG trigger) approximately 36 hr before the scheduled egg-retrieval procedure to induce ovulation. Measurements of serum FSH and E2, and details of egg retrieval have been previously described (Mok-Lin et al. 2010). Patients underwent one of three IVF treatment protocols: *a*) luteal-phase GnRH agonist protocol using low-, regular-, or high-dose leuprolide (Lupron) with pituitary desensitization begun in the luteal phase; *b*) follicular-phase GnRH-agonist/Flare protocol, in which Lupron was begun on day 2 of the follicular phase at 20 units and decreased to the standard dose of 5 units on day 5; and *c*) GnRH-antagonist protocol, in which GnRH-antagonist was begun when the lead follicle reached 14 mm in size and/or E2 levels were ≥ 1,000 pg/mL. The antagonist and flare protocols were primarily for poor responders. The flare protocol is indicated for women > 40 years of age with very poor ovarian response (i.e., inadequate follicle recruitment after controlled ovarian stimulation with gonadotropins and low peak E2 level at time of hCG trigger), whereas the antagonist protocol is used in women < 40 years of age with diminished ovarian reserve and poor ovarian response.

Endometrial thickness (millimeters) was measured by transvaginal ultrasound scan (7.5 MHz frequency; GE Logiq 3/5; GE Healthcare, Waukesha, WI) before the administration of hCG, which corresponded to 36 hr before the egg-retrieval procedure. Women with endometrial thickness < 7 mm typically did not undergo a transfer and their embryos were frozen. Following the day of egg retrieval, patients were prescribed progesterone (P), usually by intramuscular administration (50 mg/day). Nine days after egg retrieval, patients began transdermal E2 (Vivelle patches; Novartis Pharmaceuticals Corp., East Hanover, NJ) at a dose of 0.2 mg every other day. Both P and E2 were administered to hormonally prime the endometrium for embryo transfer.

Embryos were evaluated by an embryologist and selected for uterine transfer on day 2, 3, or 5 of embryo maturation in culture. A day-2 transfer was performed when the patient had only one or two embryos for transfer. Because there were few day-2 transfers (*n* = 13 IVF cycles) and they represent patients with poorer expected outcomes, they were excluded from these analyses and are not included in the 180 cycles. Implantation failure was defined as a serum β-hCG level < 6 mIU/mL typically measured 17 days (97% measured by day 17, range 15–20 days) after egg retrieval.

*Urine sample collection and urinary bisphenol A concentrations.* The 137 enrolled women provided ≤ 2 spot urine samples per IVF cycle, the first collected between cycle day 3 and day 9 of the treatment cycle, and the second on the day of the egg-retrieval procedure, typically before the procedure. Urine was collected in a sterile clean polypropylene specimen cup. Specific gravity (SG) was measured at room temperature using a handheld refractometer (National Instrument Co. Inc., Baltimore, MD) calibrated with deionized water before each measurement. The urine was divided into aliquots and frozen and stored at –80°C. Samples were shipped on dry ice overnight to the CDC where they were stored at ≤ –40°C until analysis.

The urinary concentration of free and conjugated BPA species (total BPA) was measured using online solid phase extraction (SPE) coupled to isotope dilution–high performance liquid chromatography (HPLC)–tandem mass spectrometry (MS/MS) as described before (Ye et al. 2005). First, 100 µL of urine was treated with β-glucuronidase/sulfatase (*Helix pomatia*, H1; Sigma–Aldrich, St. Louis, MO) to hydrolyze the BPA-conjugated species. BPA was then retained and concentrated on a C18 reversed-phase-size-exclusion SPE column (Merck KGaA, Darmstadt, Germany), separated from other urine matrix components using a pair of monolithic HPLC columns (Merck KGaA), and detected by negative ion–atmospheric pressure chemical ionization–MS/MS. The limit of detection (LOD) for BPA was 0.4 µg/L. In addition to study samples, each analytical run included low-concentration and high-concentration quality control materials, prepared with spiked pooled human urine, and reagent blanks to assure the accuracy and reliability of the data (Ye et al. 2005). BPA concentrations < LOD were assigned a value equal to the LOD divided by _√_^–^2 (Hornung and Reed 1990) before adjustment for urine dilution by SG, as described previously (Meeker et al. 2010).

*Statistical analysis*. Characteristics of the women and IVF cycles were summarized using means, standard deviations, and percentages, as appropriate. The geometric mean of the SG-adjusted BPA concentrations from two spot–urine samples collected during each IVF cycle was used as a measure of cycle-specific urinary BPA concentration. The distribution of these geometric mean BPA concentrations was summarized using percentiles.

Multivariate generalized estimating equation (GEE) models for repeated measures were used to evaluate the association between cycle specific urinary SG-adjusted BPA concentrations and potential risk factors for implantation failure. We used an autoregressive correlation structure to account for correlation between outcomes across repeated IVF cycles within the same woman. We modeled SG-adjusted urinary BPA concentrations in quartiles. Age (≥ 37 years or < 37 years), and day of embryo transfer (day 5 vs. day 3) were retained in the final model because of their biological and clinical relevance as indicated in previous studies (Giorgetti et al. 1995; Rehman et al. 2007).

Other variables considered as potential confounders included the number of embryos transferred (single vs. multiple); IVF protocol type (flare/antagonist vs. regular luteal phase protocol); day-3 serum FSH level (international units per liter, a measure of ovarian reserve); peak serum E2 (this corresponded to the last serum E2 measurement, 2 days before egg retrieval); endometrium thickness (< 9 mm vs. ≥ 9 mm); smoking (ever vs. never smoker); and body mass index (BMI) [overweight/obese (BMI ≥ 25 kg/m^2^) vs. normal/underweight (BMI < 25 kg/m^2^)]. Covariates that predicted implantation failure with *p* < 0.2 in univariate models and present in ≥ 5% of the cohort were evaluated for inclusion in the multivariate model using backward selection, and they were retained in the final model if their *p* was ≥ 0.10 or if the effect estimate changed by > 10% when the covariates were removed. A test for trend was performed to determine if there was a linear dose–response relationship between quartiles of urinary BPA and implantation failure. The trend test was performed by modeling the BPA quartiles as an ordinal categorical variable, using the integer values 0, 1, 2, and 3 for BPA quartiles 1, 2, 3, and 4, respectively.

As an alternative to the GEE model approach based on a logistic link, log binomial models for the relative risk were also fit. Because these models did not always converge, but yielded similar results when they did, they are not presented here. In addition, analyses restricted to first cycles only were also performed.

Because the choice of IVF treatment protocol used in a given cycle is determined by the patient’s anticipated probability of success based in part on past implantation failures and because past urinary BPA concentrations may be associated with past implantation failures and correlated with current BPA concentrations, we considered protocol type as a potential intermediate on the causal pathway between urinary BPA concentration and implantation failure. We therefore present model results both adjusted and unadjusted for treatment protocol. We also conducted a stratified analysis by type of treatment protocol. All data analyses were performed using SAS version 9.2 (SAS Institute Inc., Cary, NC).

## Results

The 137 women included in this analysis were on average 35.8 years of age, 87% were Caucasian, and < 5% were current smokers (Table 1). Approximately one-third of the couples had a primary SART diagnosis of female infertility, a third had male factor infertility, and a third had unexplained infertility. Women who had ever had a treatment cycle that resulted in an implantation failure were on average 2 years older than those who had never experienced an implantation failure. The primary treatment protocol for the 180 IVF cycles undergone by the 137 study participants was the luteal phase GnRH agonist protocol (61% of cycles; Table 2). Only 13% of the embryo transfers were single embryo transfers, and one third of the transfers were on day 5 at the blastocyst stage.

**Table 1:** Subject demographics and infertility diagnoses among 137 women undergoing IVF [n (%)].

Implantation failure since entry to study		
Characteristic	Total (n = 137)	Ever had (n = 43)	Never had (n = 94)
Age [years; mean ± SD (range)]		35.8 ± 4.0 (21 – 44)		37.0 ± 4.8 (21 – 44)		35.2 ± 3.5 (28 – 43)
< 37		79 (58)		17 (40)		62 (66)
≥ 37		58 (42)		26 (60)		32 (34)
BMIa [kg/m2; mean ± SD (range)]		24.7 ± 5.0 (16.5 – 42)		26.2 ± 5.5 (17.5 – 42.3)		23.9 ± 4.6 (16.5 – 39.1)
≥ 25		45 (33)		18 (42)		27 (29)
Race						
White		119 (87)		35 (81)		84 (89)
Black/African American		5 (4)		2 (5)		3 (3)
Asian		8 (6)		2 (5)		6 (6)
Hispanic/other		5 (4)		4 (9)		1 (1)
Smoking						
Never smoker		98 (72)		32 (74)		66 (70)
Ever smoker		39 (28)		11 (26)		28 (30)
Current smoker		5 (4)		2 (5)		3 (3)
Former smoker		34 (25)		9 (21)		25 (27)
SART diagnosisb						
Female factor infertility		49 (36)		20 (46)		29 (31)
Diminished ovarian reserve		13 (26)		7 (35)		6 (21)
Ovulation disorders		14 (29)		2 (10)		12 (41)
Endometriosis		11 (22)		6 (30)		5 (17)
Uterine disorders		1 (2)		0 (0)		1 (3)
Tubal factor		10 (20)		5 (25)		5 (17)
Male factor infertility		49 (36)		13 (30)		36 (38)
Unexplained		36 (26)		7 (16)		29 (31)
Otherc		3 (2)		3 (7)		0 (0)
Due to rounding, percentage totals may not sum to 100%. aBMI missing for 1 subject from implantation group. bPrimary diagnosis of infertility. cOther SART diagnoses: balanced translocation, Canavan’s disease, irregular antibodies.						

**Table 2:** Treatment protocols, cycle characteristics, and pregnancy outcomes for 180 IVF cycles among 137 women [n (%)].

Characteristic	IVF cyles	Implantation failure (n = 75)	Implantation (n = 105)
IVF protocol						
Luteal phase		109 (61)		33 (44)		76 (72)
Low-dose leuprolide lupron		102 (57)		31 (41)		71 (68)
Regular-dose leuprolide lupron		7 (4)		2 (3)		5 (5)
Flare		47 (26)		27 (36)		20 (19)
Antagonist		24 (13)		15 (20)		9 (9)
Day 3 FSH (IU/L)						
Mean ± SD		7.3 ± 2.3		7.6 ± 2.3		7.1 ± 2.3
Range		1.0–15.2		1–15.2		1–15.2
Peak E2 (pg/mL)a						
Mean ± SD		2,051 ± 819		2,040 ± 874		2,060 ± 783
Range		551–4,455		551–4,126		635–4,455
Endometrial thicknessa						
Thin (≤ 8 mm)		35 (19)		16 (21)		19 (18)
Mean ± SD		10.3 ± 2.0		10.1 ± 1.9		10.4 ± 2.1
Range		6–19		7–15		6–19
Day of embryo transfer						
3		120 (67)		57 (76)		63 (60)
5		60 (33)		18 (24)		42 (40)
No. of embryos transferred						
1		24 (13)		11 (15)		13 (12)
2		109 (61)		39 (52)		70 (67)
≥ 3		47 (26)		25 (33)		22 (21)
Due to rounding, percentage totals may not equal 100%. aMissing 10 peak E2 and endometrial thicknesses; the mean number of cycles was 1.3 cycles/woman.						

The 325 urine samples provided by the 137 women had urinary BPA concentrations that were comparable to general population concentrations from NHANES participants. The geometric mean was 1.53 μg/L compared with 1.97 μg/L for females in NHANES 2007–2008 (CDC 2011) (Table 3). Two urine samples were collected in 80% (144/180) of cycles. No significant differences (at *p* < 0.05) in baseline characteristics were noted between women who contributed one sample as compared with two urine samples during their treatment cycle (data not shown). For the 20% of cycles with one urine sample, the BPA concentration for that single urine sample was used as the cycle-specific urinary BPA concentration. Detectable concentrations of BPA were measured in 88% (287/325) of samples (Table 3).

**Table 3:** Distribution of cycle-specific urinary BPA concentrations (µg/L) among 137 women undergoing 180 IVF cycles (325 urine samples).

Percentile										
Detection rate	GM (SD)	Min	10th	25th	50th	75th	90th	95th	Max
BPA		88%		1.53 (2.22)		< LOD		0.60		0.89		1.50		2.40		3.76		6.04		22.07
SG-adj BPA				2.56 (1.87)		< LOD		1.27		1.69		2.33		3.79		5.91		7.82		26.48
Abbreviations: < LOD, below limit of detection (0.4 µg/L); Max, maximum; Min, minimum; SG-adj BPA, specific-gravity–adjusted BPA concentrations. All values below LOD were assigned a value equal to the LOD divided by √–2. Nine (5%) cycle-specific BPA concentrations were < LOD and are included in the percentiles. Thirty-eight (12%) individual urine samples had BPA concentrations < LOD. There was an 88% detection rate for all individual urine samples [SG range (1.001–1.035)].																				

In unadjusted logistic regression models, women ≥ 37 years of age had more than twice the odds of implantation failure as compared with women < 37 years of age, and women on a flare or antagonist protocol had a more than three times the odds of implantation failure as compared with women on the luteal phase protocol (Table 4). In IVF cycles in which the embryo transfer occurred on day 5, there was a 50% decreased odds of implantation failure compared with a day-3 embryo transfer. Day-3 FSH (international units per liter) was marginally associated with increased odds of implantation failure, but showed no association after adjustment for other covariates (age, day of embryo transfer, and IVF protocol type) in the multivariate model. The remaining covariates were not significantly associated with implantation on univariate analyses (*p* < 0.2) and therefore did not qualify for inclusion in our multivariate model. Urinary BPA concentrations were significantly higher in women who underwent a low responder treatment protocol as compared with the regular luteal phase protocol (4.08 vs. 2.61 µg/L, *p* < 0.001). BPA concentrations did not vary significantly by levels of other covariates of interest that were considered for inclusion in the multivariable model.

**Table 4:** Crude and adjusted ORs (95% CIs) for associations of quartiles of urinary BPA concentrations with implantation failure among 137 women undergoing 180 IVF cycles.

Characteristic	n	Unadjusted	p-Value	Adjusteda	IVF-protocol adjustedb
SG-adjusted BPA quartiles (range µg/L)										
1 (≤ 1.69)		45		Reference				Reference		
2 (1.70–2.33)		45		1.01 (0.38, 2.66)				1.02 (0.35, 2.95)		1.05 (0.36, 3.03)
3 (2.34–3.79)		46		1.57 (0.70, 3.52)				1.60 (0.70, 3.78)		1.52 (0.65, 3.55)
4 (3.80–26.48)		44		2.38 (0.97, 5.83)				2.11 (0.84, 5.31)		1.90 (0.73, 4.96)
p-Trend				0.03				0.06		0.12
Age (≥ 37 vs. < 37 years)				2.22 (1.15, 4.28)		0.02		1.89 (0.97, 3.65)		1.50 (0.76, 2.95)
Embryo transfer day (day 5 vs. day 3)				0.48 (0.24, 0.95)		0.03		0.53 (0.26, 1.08)		0.68 (0.32, 1.45)
IVF protocol (flare/antagonist vs. luteal)				3.16 (1.70, 5.87)		< 0.001				2.09 (1.03, 4.24)
No. embryos transferred (1, ≥ 2)				0.67 (0.29, 1.54)		0.35				
Day-3 FSH (IU/L)				1.87 (0.95, 1.28)		0.19				
Peak E2 (pg/mL)				1.00 (0.99, 1.00)		0.68				
Endometrial thickness (≤ 8 mm vs. > 8 mm)				1.50 (0.69, 3.24)		0.30				
Ever smoker vs. never smoker				1.09 (0.55, 2.16)		0.80				
BMI (≥ 25 kg/m2 vs. < 25 kg/m2)c				1.35 (0.69, 2.64)		0.38				
Urine collection year (continuous)				0.84 (0.56, 1.27)		0.42				
aAdjusted for age [≥ 37 years vs. < 37 years (referent group)] and day of embryo transfer [day 5 vs. day 3 (referent group)]. bAdjusted for age (≥ 37 years vs. < 37 years), day of embryo transfer (day 5 vs. day 3), and IVF protocol [flare/antagonist vs. luteal (referent group)]. cOne missing value.										

**Figure 1:**
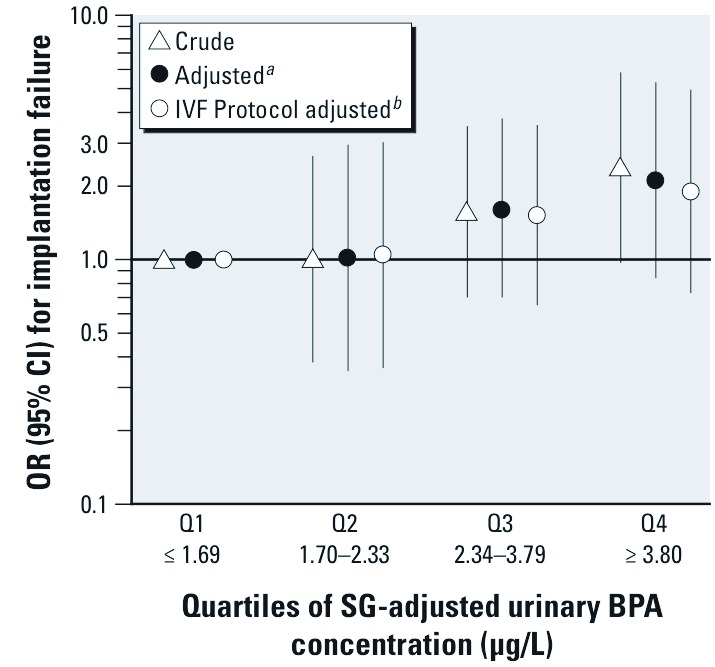
Crude and adjusted OR (95% CI) for the associations of quartiles (Q) of urinary BPA concentrations in relation to implantation failure among 137 women undergoing 180 IVF cycles. ***^a^***Adjusted for age (≥ 37 years vs. < 37 years) and day of embryo transfer (day 5 vs. day 3). ***^b^***Adjusted for age (≥ 37 years vs. < 37 years), day of embryo transfer (day 5 vs. day 3), and IVF protocol (flare/antagonist vs. luteal).

In the unadjusted model, the odds of implantation failure increased linearly with increasing quartiles of urinary BPA concentrations: odds ratios (OR) [95% confidence interval (CI)] were 1.01 (0.38, 2.66), 1.57 (0.70, 3.52), and 2.38 (0.97, 5.83) for quartiles 2, 3, and 4, respectively, compared with the lowest quartile (*p*-trend = 0.03) (Table 4 and Figure 1). Results were similar after adjusting for older age (> 37 years) and day of embryo transfer (day 5 vs. day 3), although slightly attenuated for the fourth versus first quartile of exposure (*p*-trend = 0.06). Finally, after adjustment for IVF protocol, the associations were further attenuated but remained consistent with a positive linear dose–response relationship.

In models stratified by IVF protocol, there was a stronger association of urinary BPA concentration with implantation failure among women undergoing flare/antagonist protocols (*n* = 51 women, 71 cycles) compared with women undergoing the luteal protocol (*n* = 95 women, 109 cycles). Nine women were in the luteal protocol group for one cycle and the flare/antagonist group for a subsequent cycle. Among women in the flare/antagonist protocol, the ORs (95% CI) for implantation failure by quartile of urinary BPA concentration, after adjustment for age (years) and day of embryo transfer (day 5 vs. day 3), were 1.50 (0.26, 8.58), 2.12 (0.47, 9.62), and 3.59 (0.74, 17.43) for quartiles 2, 3, and 4, respectively, compared with the lowest quartile (*p*-trend = 0.07). In contrast, corresponding estimates for women in the luteal protocol group were 0.68 (0.13, 3.55), 1.02 (0.35, 2.95), and 1.04 (0.36, 3.04) for quartiles 2, 3, and 4, respectively, compared with the lowest quartile (*p*-trend = 0.83). Women treated with the alternative flare or antagonist protocols were more likely than women treated with the luteal protocol to have had past IVF treatment cycle failures (73% vs. 34%), diminished ovarian reserve (mean day-3 FSH 8.8 IU/L vs. 6.6 IU/L), and diminished ovarian response to controlled ovarian FSH hyperstimulation (mean peak serum E2 1,735 pg/mL vs. 2,258 pg/mL, mean number of eggs retrieved 7.6 vs. 12.7). Finally, women on the alternative protocol were almost 4 years older than women on the luteal phase protocol (mean age 38.1 years vs. 34.3 years).

## Discussion

In the present study, the association between urinary BPA concentrations and implantation failure increased with increasing urinary BPA quartiles after adjusting for age and day of embryo transfer. Women in the fourth quartile of exposure (SG-adjusted urinary BPA, 3.80–26.48 μg/L) had almost twice the odds of implantation failure as women in the first quartile of exposure (≤ 1.69 μg/L). This trend remained after adjusting for IVF protocol.

After stratifying by protocol type, we identified a potentially more sensitive subgroup of women. There was a stronger association between urinary BPA concentrations and implantation failure among women undergoing the flare or antagonist protocol (alternative/low responder protocol) as compared with women undergoing the luteal protocol, though numbers were small within strata and there was considerable overlap in 95% CI for corresponding estimates between the two groups. Women on the flare/antagonist protocol were older with diminished ovarian reserve and response than women on the luteal protocol. Although speculative, diminished ovarian reserve may impart increased sensitivity to implantation failure in relation to BPA exposure.

Implantation in animals and humans is hormonally modulated by E2 and P via regulation of expression of endometrial proteins that play an important role in endometrial receptivity at the time of implantation (Cooke et al. 1997). Increased secretion of E2 in the preovulatory phase is necessary to stimulate the proliferation and differentiation of the uterine epithelial cells. In the luteal phase, continued production of P stimulates the proliferation and stimulation of the stromal cells, thus preparing the endometrium for successful embryo implantation (Norwitz et al. 2001). E2 and P also play a role in embryo development and embryo migration through the oviduct, though the latter is less relevant in women undergoing IVF because eggs are retrieved from the ovary and cultured embryos are transferred directly into the uterine cavity, bypassing the oviduct. At the level of the embryo, E2 plays an important role in the activation of the dormant blastocyst, which is critical for successful implantation (Paria et al. 1993). Previous findings from the study cohort indicated that higher urinary BPA concentrations were associated with decreased peak serum E2 levels and decreased number of eggs retrieved in women undergoing IVF (Mok-Lin et al. 2010).

Evidence from animal studies suggests that BPA’s estrogenic properties may disrupt implantation via two mechanisms (Berger et al. 2007, 2008, 2010; Varayoud et al. 2011): *a*) accelerating the rate of blastocyst development and thus leading to mismatch in timing with the appropriate uterine receptivity window (Takai et al. 2001), and *b*) directly decreasing uterine receptivity to blastocyst implantation (Berger et al. 2010). Environmentally relevant doses or low concentrations (1–3 nM) of BPA have been shown to significantly increase the developmental rates of two-cell mouse embryos to blastocysts. At a high concentration (100 mM), BPA significantly decreased the *in vitro* developmental rates of the embryos (Takai et al. 2000). This could result in an embryo uterine mismatch and impair successful implantation.

In a recent study, high doses of BPA administered subcutaneously (3.375, 6.75, and 10.125 mg/day) to mice during the peri-implantation phase resulted in a dose-dependent increase in luminal area (i.e., altered endometrial morphology) and decreased expression of estrogen receptor α (ERα) and progesterone receptor (PR) via BPA action on the ER and decreased PR expression leading to implantation failure (Berger et al. 2010). Timing of exposure was critical, and a single 6.75-mg dose of subcutaneously administered BPA on day 0 or 10.125 mg on day 1 of pregnancy was sufficient to significantly reduce the number of implantation sites in mice. However, when the same dose was administered on day 2 of pregnancy, it had no effect on the number of implantation sites observed (Berger et al. 2008). In contrast to humans, in mice, implantation can occur as late as day 4 of pregnancy because blastocysts can remain in a state of quiescence until uterine conditions are optimal for embryo implantation (Carson et al. 2000). A previous study by Berger and colleagues found that an average of 68.84 mg of BPA ingested per day (substantially higher than typical daily doses to which humans are exposed) resulted in a complete block of implantation, whereas comparable results were achieved with a dose of only 6.75 mg administered via subcutaneous administration (Berger et al. 2007). The most common route of human exposure to BPA is through ingestion (Vandenberg et al. 2007).

A recent study in rats showed that BPA at doses as low as 0.05 mg/kg/day [the U.S. Environmental Protection Agency (EPA) oral reference dose (U.S. EPA 1993)] resulted in decreased Hoxa 10 expression, an essential transcription factor required for endometrial receptivity and implantation (Varayoud et al. 2011). Down-regulation of the ERα and PR was also observed, accompanied by a significantly decreased number of implantation sites.

One potential limitation of the present study is that there is within-individual variability in urinary BPA concentrations and collecting single spot urine samples may result in exposure misclassification (Braun et al. 2011; Ye et al. 2011). However, a previous study in our cohort showed that a single urine sample had moderate sensitivity for predicting an individual’s longer-term exposure over several weeks or months (Mahalingaiah et al. 2008). The sensitivity of classifying a subject in the highest tertile using a single urine sample was 0.64. In the present study, during each cycle, urine samples were collected twice over a period of 2 weeks. The Spearman correlation of within-cycle urinary BPA concentrations for this short time interval was 0.18. Although two measures of urinary BPA concentration are better than a single spot sample at classifying BPA exposure, we cannot be certain that we collected samples within the relevant window of exposure given the short half-life of BPA (Volkel et al. 2002) and its high variability over time. The exact timing of urine collection was dependent on the date and time of medical appointments and egg-retrieval procedures and were not necessarily first morning or fasting urines.

Another potential limitation is uncertainty regarding the generalizability of the results to women who conceive naturally. It is possible that women undergoing IVF may be more sensitive to BPA exposure for a variety of reasons, including their underlying infertility, the *in vitro* conditions of early embryonic development, or the ovarian hyperstimulation protocols. Despite uncertainty regarding generalizability to women conceiving naturally, using IVF as a model to study human reproduction has several strengths. This includes the assessment of early developmental end points that are not observable in women conceiving naturally but have been shown to be susceptible to environmental chemical exposure in experimental studies (Berger et al. 2007, 2008, 2010; Hunt et al. 2003; Takai et al. 2000). Furthermore, because approximately 10–15% of the population in the United States and developed countries are infertile (Fritz and Speroff 2011), the results of the present study may be generalizable to a large portion of the general population. Additional strengths include the preconception prospective study design, allowing us to collect exposure information before the outcome of interest and performing accurate clinical ascertainment of implantation failure. One of the limitations of conventional epidemiologic designs in naturally conceiving women is that it is logistically challenging and expensive to collect and analyze daily urines for multiple months to assess implantation failure (Wilcox et al. 1988).

Finally, by applying a GEE approach to account for correlated outcomes across cycles within a given woman, we were able to optimize all the data available to us, which is particularly important given the relatively small size of our sample population. Alternative modeling approaches such as nonlinear mixed effect models may be preferable, particularly given the potential for ORs to overestimate relative risks for common outcomes (e.g., the 42% implantation failure in our study population), but they could not be applied given the limited sample size. However, results based on only first cycle and on log binomial models were consistent with those presented.

As recruitment continues and our study population increases, we plan to extend these analyses to explore the contribution of the male gametes and male exposures to BPA to implantation failure. We also plan to explore the association of maternal and paternal BPA exposure with embryo quality and cleavage rate to further understand the potential affect of BPA on implantation and fertility.

In conclusion, these preliminary findings are, to the best of our knowledge, the first human data on the association between environmental exposure to BPA and implantation failure. The results suggest a positive linear dose–response association between urinary BPA concentrations and implantation failure among women undergoing IVF treatment.
